# *Kingella kingae* PilC1 and PilC2 are adhesive multifunctional proteins that promote bacterial adherence, twitching motility, DNA transformation, and pilus biogenesis

**DOI:** 10.1371/journal.ppat.1010440

**Published:** 2022-03-30

**Authors:** Alexandra L. Sacharok, Eric A. Porsch, Taylor A. Yount, Orlaith Keenan, Joseph W. St. Geme

**Affiliations:** 1 University of Pennsylvania Perelman School of Medicine, Philadelphia, Pennsylvania, United States of America; 2 Department of Pediatrics, Children’s Hospital of Philadelphia, Philadelphia, Pennsylvania, United States of America; Northwestern University Feinberg School of Medicine, UNITED STATES

## Abstract

The gram-negative bacterium *Kingella kingae* is a leading cause of osteoarticular infections in young children and initiates infection by colonizing the oropharynx. Adherence to respiratory epithelial cells represents an initial step in the process of *K*. *kingae* colonization and is mediated in part by type IV pili. In previous work, we observed that elimination of the *K*. *kingae* PilC1 and PilC2 pilus-associated proteins resulted in non-piliated organisms that were non-adherent, suggesting that PilC1 and PilC2 have a role in pilus biogenesis. To further define the functions of PilC1 and PilC2, in this study we eliminated the PilT retraction ATPase in the Δ*pilC1*Δ*pilC2* mutant, thereby blocking pilus retraction and restoring piliation. The resulting strain was non-adherent in assays with cultured epithelial cells, supporting the possibility that PilC1 and PilC2 have adhesive activity. Consistent with this conclusion, purified PilC1 and PilC2 were capable of saturable binding to epithelial cells. Additional analysis revealed that PilC1 but not PilC2 also mediated adherence to selected extracellular matrix proteins, underscoring the differential binding specificity of these adhesins. Examination of deletion constructs and purified PilC1 and PilC2 fragments localized adhesive activity to the N-terminal region of both PilC1 and PilC2. The deletion constructs also localized the twitching motility property to the N-terminal region of these proteins. In contrast, the deletion constructs established that the pilus biogenesis function of PilC1 and PilC2 resides in the C-terminal region of these proteins. Taken together, these results provide definitive evidence that PilC1 and PilC2 are adhesins and localize adhesive activity and twitching motility to the N-terminal domain and biogenesis to the C-terminal domain.

## Author summary

*Kingella kingae* is an emerging pediatric pathogen that is a leading cause of osteoarticular infections in children under the age of four. Adherence to epithelial cells is thought to be the first step in *K*. *kingae* colonization of the host and a prerequisite for invasive disease. Previous work has established that type IV pili are responsible for *K*. *kingae* adherence to host cells. In this work we identify the *K*. *kingae* pilus adhesins and localize the adhesive region to the N-terminal domain of these two proteins. We further establish that the two adhesins have distinct binding specificities and also influence other biologic processes. Our study provides new insights into the adherence mechanisms of an increasingly recognized pediatric pathogen and furthers our understanding of *K*. *kingae* interactions with host cells, identifying new potential therapeutic targets.

## Introduction

*Kingella kingae* is a gram-negative bacterium that colonizes the upper respiratory tract in young children [1]. *K*. *kingae* colonization is a common occurrence, with approximately 70% of children being colonized by 48 months of age and approximately 10% of young children carrying this bacterium in the oropharynx at any given time [2,3]. While *K*. *kingae* is usually a commensal organism, on occasion it breaches the epithelial barrier, enters the bloodstream, and spreads hematogenously to sites of infection, causing invasive diseases such as septic arthritis and osteomyelitis [4]. Recent advances in molecular diagnostics have identified *K*. *kingae* as a leading cause of osteoarticular infections in children between 6 months and 4 years of age [5,6].

Adherence to epithelial cells is believed to be the first step in *K*. *kingae* colonization of the oropharynx and a prerequisite for invasive disease. *K*. *kingae* adherence to host cells is a two-step process. The initial step is mediated by type IV pili (T4P), which then retract, bringing the bacterium closer to epithelial cells [7,8]. Retraction displaces the polysaccharide capsule, allowing a surface expressed adhesin called the *Kingella* NhhA homolog (Knh) to mediate the second step required for full-level adherence [8,9]. Strains that lack retractile T4P exhibit an intermediate level of adherence, due to the lack of Knh-mediated adherence [8].

The *K*. *kingae* PilC1 and PilC2 pilus-associated proteins promote piliation and T4P adhesive activity, with expression of at least one of these proteins being required for piliation and adherence to epithelial cells *in vitro* [7]. These proteins have homologs in other bacterial species, including PilY1 in *Pseudomonas aeruginosa*, PilY1 in *Legionella pneumophila*, and PilC1 and PilC2 in *Neisseria gonorrhoeae* and *Neisseria meningitidis* (constituting the “PilC family”). These PilC family proteins have been shown to play a role in pilus assembly, pilus-mediated adherence, and pilus-mediated twitching motility [10–14]. Previous observations by Morand *et al*. showed that the N-terminal portion of the *N*. *meningitidis* PilC1 protein was required for PilC1-mediated bacterial adherence in engineered *N*. *meningitidis* strains [15]. Additionally, in studies of the *N*. *gonorrhoeae* PilC1 protein, Cheng and colleagues found that full-length recombinant PilC1 was able to block *N*. *gonorrhoeae* adherence to cultured epithelial cells, while the truncated PilC1 C-terminal domain had no blocking activity [16]. Because *K*. *kingae* PilC1 and PilC2 are required for surface piliation, it is unclear whether these proteins possess inherent adhesive activity.

The *K*. *kingae* PilC1 and PilC2 proteins share limited homology with each other, with only 7% identity and 16% similarity overall [7], contrasting with the other known PilC-containing systems, which possess only one protein as in *P*. *aeruginosa* and *L*. *pneumophila* or two highly homologous proteins as in *N*. *gonorrhoeae* and *N*. *meningitidis*. Interestingly, in previous work we found that while both *K*. *kingae* PilC1 and PilC2 were able to promote twitching motility, *K*. *kingae* exhibited significantly different levels of twitching motility depending on which PilC protein was present, with PilC1 promoting a hyper-motile phenotype in the absence of PilC2 and with PilC2 mediating slightly reduced twitching in the absence of PilC1 [17].

In the present study we examined the specific functions of the *K*. *kingae* PilC1 and PilC2 proteins. We demonstrate that PilC1 and PilC2 are adhesins and have distinct binding specificities. In addition, we establish that the N-terminal domains of these proteins harbor adhesive activity and are essential for twitching motility. In contrast, the pilus assembly function resides in the C-terminal domain of these proteins.

## Results

### Elimination of the *K*. *kingae* PilT retraction ATPase restores surface piliation but not adherence in a Δ*pilC1*Δ*pilC2* mutant

**Fig 1:**
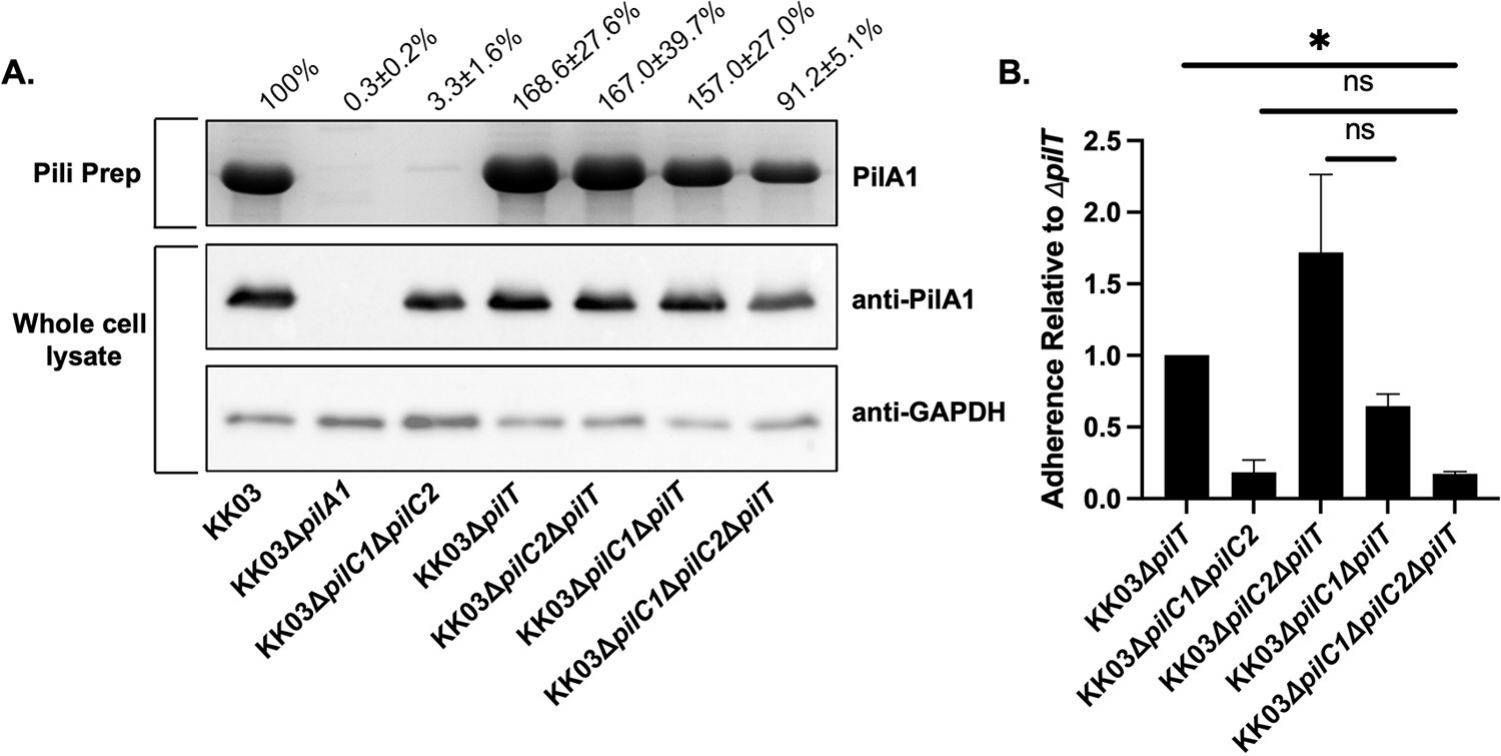
Deletion of retraction ATPase PilT restores surface piliation but not adherence in a *K*. *kingae* Δ*pilC1*Δ*pilC2* mutant strain. (A) Sheared pili fractions of strains KK03, KK03Δ*pilA1*, KK03Δ*pilC1*Δ*pilC2*, KK03Δ*pilT*, KK03Δ*pilC2*Δ*pilT*, KK03Δ*pilC1*Δ*pilT*, and KK03Δ*pilC1*Δ*pilC2*Δ*pilT* were boiled and separated using SDS-PAGE. For pili preps, the PilA1 pilin monomer band was stained with Coomassie blue. For whole cell lysates, the PilA1 pilin monomer band was detected by Western blot analysis using polyclonal antiserum GP65 [52] to PilA1. GAPDH was detected by Western blot analysis to control for total protein using polyclonal antiserum CHP-GP22. The values above the gel and blot images are densitometry measurements of the PilA1 band from the sheared pilus preparations (pili prep) gel that were normalized to the α-GAPDH band intensities and are expressed as a percentage of the band intensity of the wild type strain KK03. The densitometry values are listed ± standard error of the mean from three biological replicates, and representative gel and blot images are shown. (B) Strains KK03Δ*pilC1*Δ*pilC2*, KK03Δ*pilT*, KK03Δ*pilC2*Δ*pilT*, KK03Δ*pilC1*Δ*pilT*, and KK03Δ*pilC1*Δ*pilC2*Δ*pilT* were added to monolayers of Chang epithelial cells of HeLa origin and evaluated for adherence. Percent adherence was calculated based on the ratio of recovered bacteria to the inoculum. Error bars represent standard error of the mean, n = 3. * indicates significance of P < 0.05 as determined by a one-way ANOVA using Bonferroni correction for multiple comparisons. ns indicates no significance.

In previous work, we observed that insertional inactivation of *pilC1* and *pilC2* in *K*. *kingae* resulted in an extreme piliation defect and a non-adherent phenotype [7], suggesting that PilC1 and PilC2 are critical for pilus assembly. To determine whether elimination of PilC1 and PilC2 results in a loss of adhesive activity simply because the resulting organisms are non-piliated, we insertionally inactivated the *K*. *kingae pilT* gene in the KK03Δ*pilC1*Δ*pilC2* double knockout strain (Table 1), using strains KK03 (wild type), KK03Δ*pilA1* (lacking the major pilin subunit and thus lacking surface fibers), KK03Δ*pilT* (lacking PilT-mediated retraction), KK03Δ*pilC1*Δ*pilT* (lacking PilC1 and PilT-mediated retraction), and KK03Δ*pilC2*Δ*pilT* (lacking PilC2 and PilT-mediated retraction) as controls. As expected, the resulting mutant regained piliation to greater than 90% of the level of wild type strain KK03 as assessed by densitometry analysis of the PilA1 band in pilus preparations normalized to the GAPDH loading control (Fig 1A), consistent with previous work indicating that the *pilT* gene encodes an inner membrane ATPase that mediates pilus retraction in *K*. *kingae* and *N*. *gonorrhoeae* [8,18]. As shown in Fig 1B, while strains KK03Δ*pilC1*Δ*pilT* and KK03Δ*pilC2*Δ*pilT* were adherent in assays with Chang cells of HeLa origin, the KK03Δ*pilC1*Δ*pilC2*Δ*pilT* mutant was non-adherent (Fig 1B), supporting the possibility that PilC1 and PilC2 have inherent adhesive activity.

**Table 1:** Strains and plasmids used in this study.

Strain or Plasmid	Description	Reference or Source
*E*. *coli* strains		
DH5α	*E*. *coli* F^−^ **ϕ**80d*lacZ*Δ*M15* Δ(*lacZYA-argF*)*U169 deoR recA1 endA1 hsdR17*(r_K_^−^ m_K_^+^) *phoA supE441 thi-1 gyrA96 relA1*	[19]
XL-10 Gold	Tet^r^Δ(*mcrA*)183 Δ(*mcrCB-hsdSMR-mrr*)*173 endA1 supE44 thi-1 recA1 gyrA96 relA1 lac* Hte [F´ *proAB lacI*^q^*Z*Δ*M15* Tn*10* (Tetr) Amy Cam^r^]	Agilent
BL21(DE3) omp8	*E*. *coli* B F^−^ *dcm ompT hsdS*(r_B_^−^ m_B_^−^) *gal* λ(DE3) Δ*lamB ompF*::Tn5 *ΔompA ΔompC*	[20]
*K*. *kingae* strains		
KK03	Naturally occurring spreading and corroding variant of septic arthritis clinical isolate 269–492	[21]
KK03 derivatives		
Δ*pilA1*	KK03 with either an *aphA3* or *tetM* marked *pilA1* deletion	[7] and this work
Δ*pilF*	KK03 with an *aphA3* marked *pilF* deletion	[17]
Δ*pilC1*	KK03 with a *tetM* marked *pilC1* deletion	[17]
Δ*pilC2*	KK03 with an unmarked *pilC2* deletion	This work
Δ*pilC1*Δ*pilC2*	KK03 with a *tetM* marked *pilC1* deletion and an unmarked *pilC2* deletion	This work
Δ*pilT*	KK03 with an *ermC* marked *pilT* deletion	[17]
Δ*pilC1*Δ*pilT*	KK03 with an *ermC* marked *pilT* deletion and an *tetM* marked *pilC1* deletion	This work
Δ*pilC2*Δ*pilT*	KK03 with an *ermC* marked *pilT* deletion and an unmarked *pilC2* deletion	This work
Δ*pilC1*Δ*pilC2*Δ*pilT*	KK03 with a *tetM* marked *pilC1* deletion, an unmarked *pilC2* deletion, and an *ermC* marked *pilT* deletion	This work
Δ*pilC2*-ErmPilC1	KK03 *pilC2* deletion strain with an *ermC* insertion upstream of WT *pilC1*	[17]
Δ*pilC2*-ErmPilC1_Cterm_	KK03 Δ*pilC2*-ErmPilC1 with a deletion of the 5’-region of *pilC1*	This work
Δ*pilC1*-KanPilC2	KK03 *pilC1* deletion strain with an *aphA3* insertion immediately downstream of WT *pilC2*	[17]
Δ*pilC1*-KanPilC2_Cterm_	KK03 Δ*pilC1*-KanPilC2 with a deletion of the 5’-region of *pilC2*	This work
Plasmids		
pET22b	Protein expression vector	MilliporeSigma
pET22b/PilC1	For expression of 6xHisPilC1	This work
pET22b/PilC2	For expression of 6xHisPilC2	This work
pET22b/PilC1_Nterm_	For expression of 6xHisPilC1 N-terminal domain	This work
pET22b/PilC2_Nterm_	For expression of 6xHisPilC2 N-terminal domain	This work
pFalcon2	Source of *aphA3* kanamycin resistance gene	[22]
pHSX*tetM*4	Source of *tetM* tetracycline resistance gene	[23]
pIDN4	Source of *ermC* erythromycin resistance gene	[24]
pUC19/Δ*pilA1*:*kan*	*pilA1* deletion construct with an *aphA3* marked *pilA1* deletion	[7]
pUC19/Δ*pilA1*:*tet*	*pilA1* deletion construct with a *tetM* marked *pilA1* deletion	This work
pUC19/Δ*pilC1*	*pilC1* deletion construct with a *tetM* marked *pilC1* deletion	[7]
pUC19/Δ*pilT*	*pilT* deletion construct with an *ermC* marked *pilT* deletion	[17]
pUC19/Erm*pilC1*	For introduction of *ermC* marked *pilC1* locus	[17]
pUC19/*pilC2*Kan	For introduction of *aphA3* marked *pilC2* locus	[17]
pUC19/Erm*pilC1*ΔNterm	Construct transformed into strain Δ*pilC2* to generate strain Δ*pilC2* PilC1_Cterm_	This work
pUC19/*pilC2*ΔNtermKan	Construct transformed into strain Δ*pilC1* to generate strain Δ*pilC1* PilC2_Cterm_	This work
pTrc99A/Δ*knh*:*aphA3*	*knh* deletion construct marked with an *aphA3* kanamycin resistance marker	This work
pTrc99A/Δ*knh*:*ermC*	*knh* deletion construct marked with an *ermC* erythromycin resistance marker	This work
pBAD18	Protein expression vector with arabinose-inducible promoter	[25]
pBAD-*pilC1*	pBAD18 encoding recombinant PilC1 minus the signal peptide	This work
pHAT10	Protein expression vector for generating HAT-fusions	Takara Bio
pHAT10-*gapdh*	pHAT10 encoding the HAT-GAPDH fusion protein	This work

### *K*. *kingae* PilC1 and PilC2 proteins are adhesins with distinct protein structures

**Fig 2:**
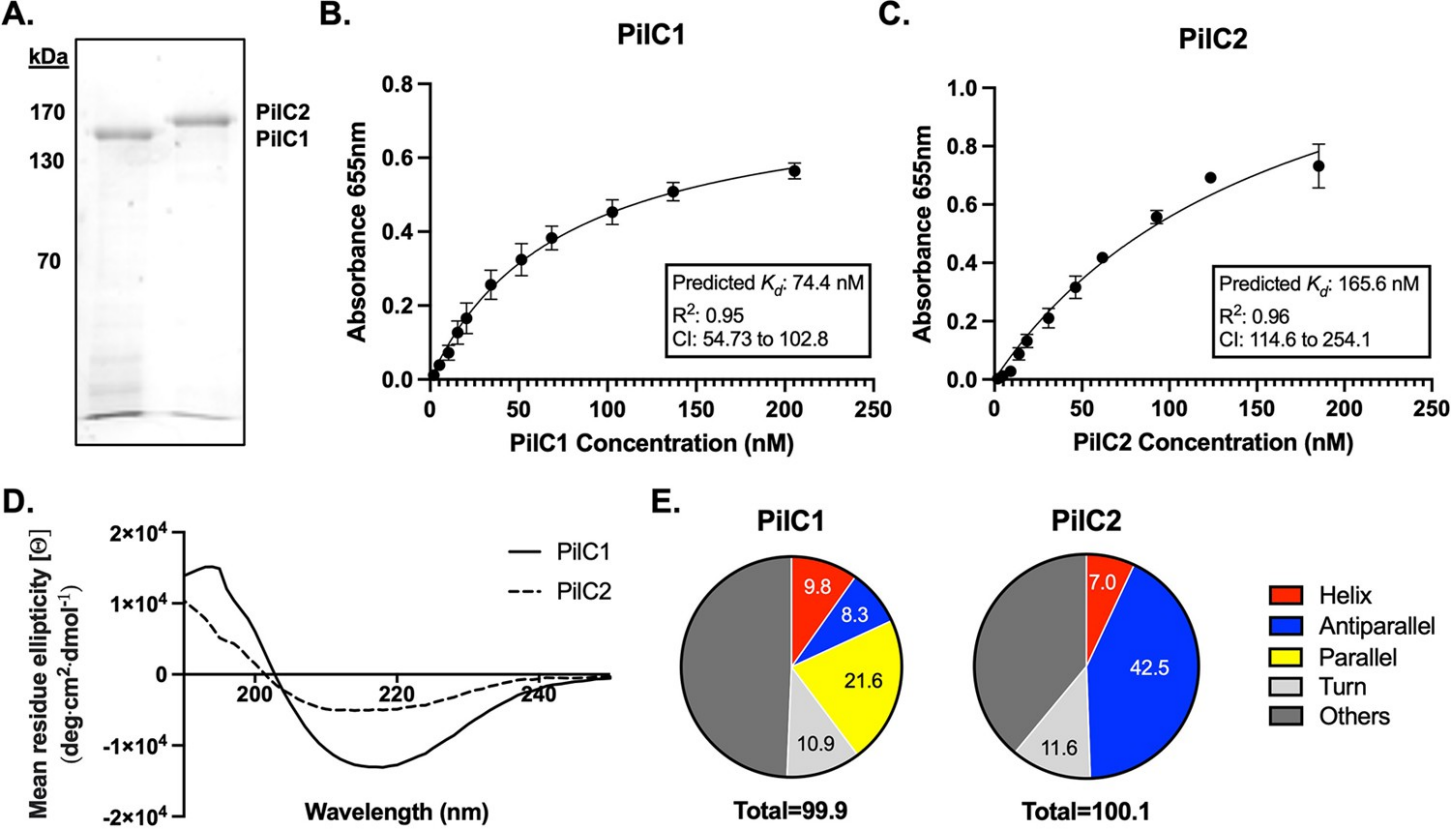
PilC1 and PilC2 are adhesins with distinct protein structures. (A) Recombinant PilC1 and PilC2 were purified and separated on a 7.5% SDS PAGE gel and stained with Coomassie blue. Proteins were added to monolayers of Chang epithelial cell of HeLa origin at increasing concentrations in 50 mM Tris-HCl, pH 8.5. (B, C) Adherence was detected by ELISA using polyclonal antiserum CHP-GP7 for PilC1 (B) or GP103 for PilC2 (C) and a secondary anti-guinea pig antibody conjugated to horseradish peroxidase (HRP). Non-linear regressions were fit using the GraphPad Prism one site specific binding model fit to total data from 3 independent runs, and the *K*_*d*_ was calculated based on the regression. Error bars represent standard error of the mean, n = 3. (D, E) Circular dichroism was carried out on purified PilC1 and PilC2 (D) and the resultant spectra were used to predict protein secondary structures (E). The CD spectra are representative graphs of three independent analyses carried out on different batches of purified protein.

To further explore the possibility that PilC1 and PilC2 are adhesins, we purified recombinant N-terminal 6X histidine-tagged PilC1 and PilC2 (Fig 2A) and examined binding to epithelial cell monolayers. As shown in Fig 2B and 2C, PilC1 exhibited saturable binding with a *K*_*d*_ of 74.4 nM, while PilC2 displayed saturable binding with a *K*_*d*_ of 165.6 nM. Examination of the purified proteins by circular dichroism (CD) spectroscopy revealed that PilC1 and PilC2 have distinct CD spectra when comparing mean residue ellipticity (Fig 2D). Based on the CD spectra, PilC1 is predicted to contain 9.8% helices, 8.3% antiparallel sheets, 21.6% parallel sheets, and 10.9% turns (Fig 2E), while PilC2 is predicted to contain 7.0% helices, 11.6% turns, 42.5% antiparallel sheets, and no parallel sheets (Fig 2E). Taken together, these data demonstrate that the *K*. *kingae* PilC1 and PilC2 proteins are adhesins and have different binding affinities and distinct global protein structures.

### *K*. *kingae* PilC1, but not PilC2, mediates bacterial adherence to extracellular matrix proteins

**Fig 3:**
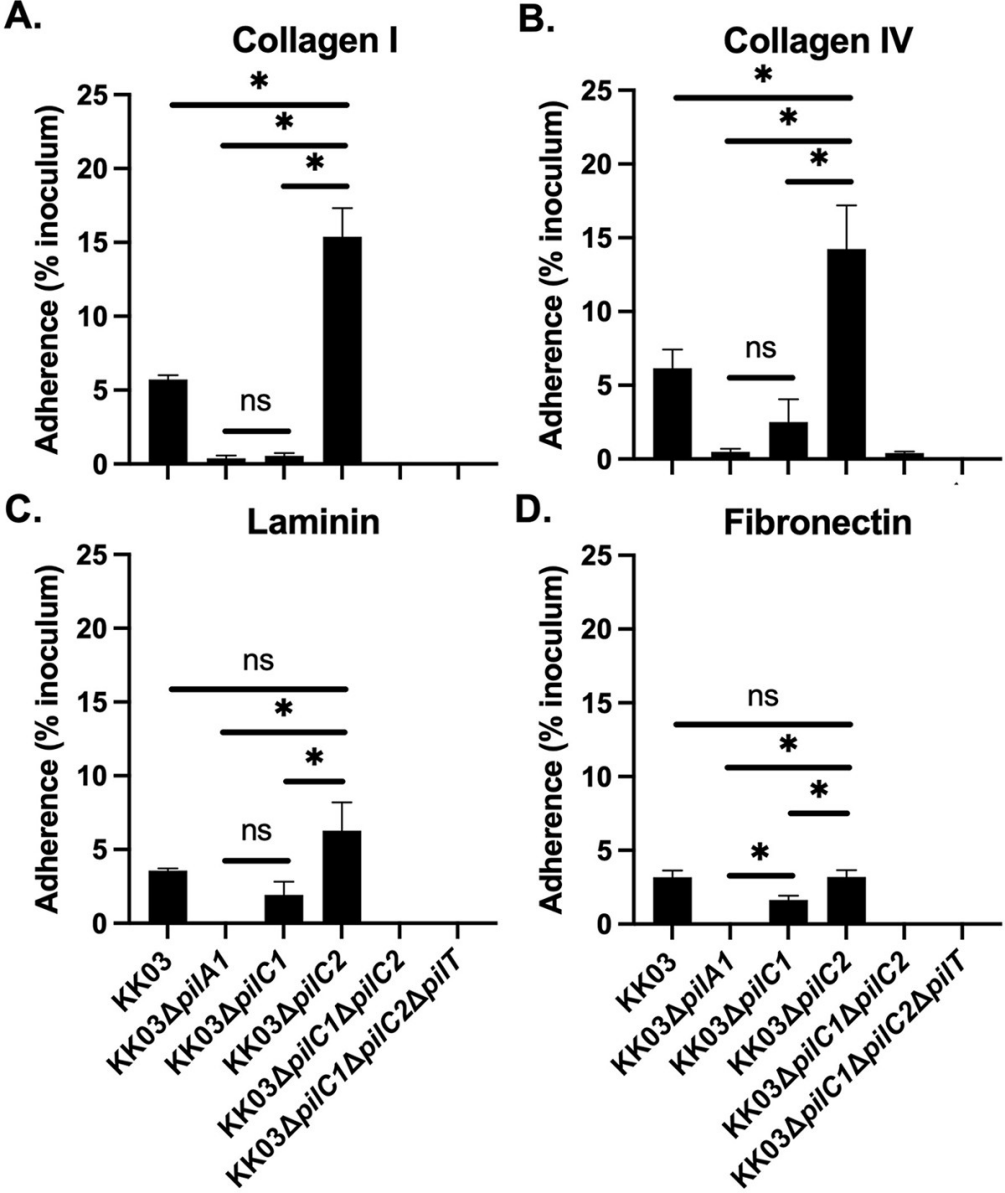
PilC1, but not PilC2, mediates *K*. *kingae* adherence to extracellular matrix. (A-D) Strains KK03, KK03Δ*pilA1*, KK03Δ*pilC1*, KK03Δ*pilC2*, KK03Δ*pilC1*Δ*pilC2*, and KK03Δ*pilC1*Δ*pilC2*Δ*pilCT* strains were added to plates coated with collagen I (A), collagen IV (B), laminin (C), or fibronectin (D) and evaluated for adherence. Percent adherence was calculated based on the ratio of recovered bacteria to the inoculum. Error bars represent standard error of the mean, n = 3. * indicates significance of P < 0.05 as determined by a one-way ANOVA using Bonferroni correction for multiple comparisons. ns indicates no significance.

Given the limited homology between PilC1 and PilC2, the observed differences in PilC1 and PilC2 binding affinities with epithelial cells, and the different PilC1 and PilC2 predicted protein structures, we wondered whether these proteins might have distinct adhesive specificities. As shown in Fig 3, WT strain KK03 showed substantial adherence to collagen I, collagen IV, laminin, and fibronectin. In contrast, the non-piliated Δ*pilA1* mutant was nonadherent to these extracellular matrix (ECM) proteins, demonstrating that adherence is pilus-mediated. To assess the role of PilC1 and PilC2, we examined the mutant strains expressing only PilC1 (KK03Δ*pilC2*) or only PilC2 (KK03Δ*pilC1*). As shown in Fig 3, these assays revealed that PilC1, but not PilC2, was capable of mediating adherence to collagen I, collagen IV, laminin, and fibronectin. Interestingly, adherence to collagen I, collagen IV, and laminin was significantly greater by strain KK03Δ*pilC2* than by the wild type strain, suggesting that PilC2 may interfere with PilC1-mediated binding. These results demonstrate that PilC1 is capable of binding to ECM proteins and establish that PilC1 and PilC2 have distinct binding specificities.

### The *K*. *kingae* PilC1 and PilC2 C-terminal domains promote production of pilus fibers that lack adhesive activity

**Fig 4:**
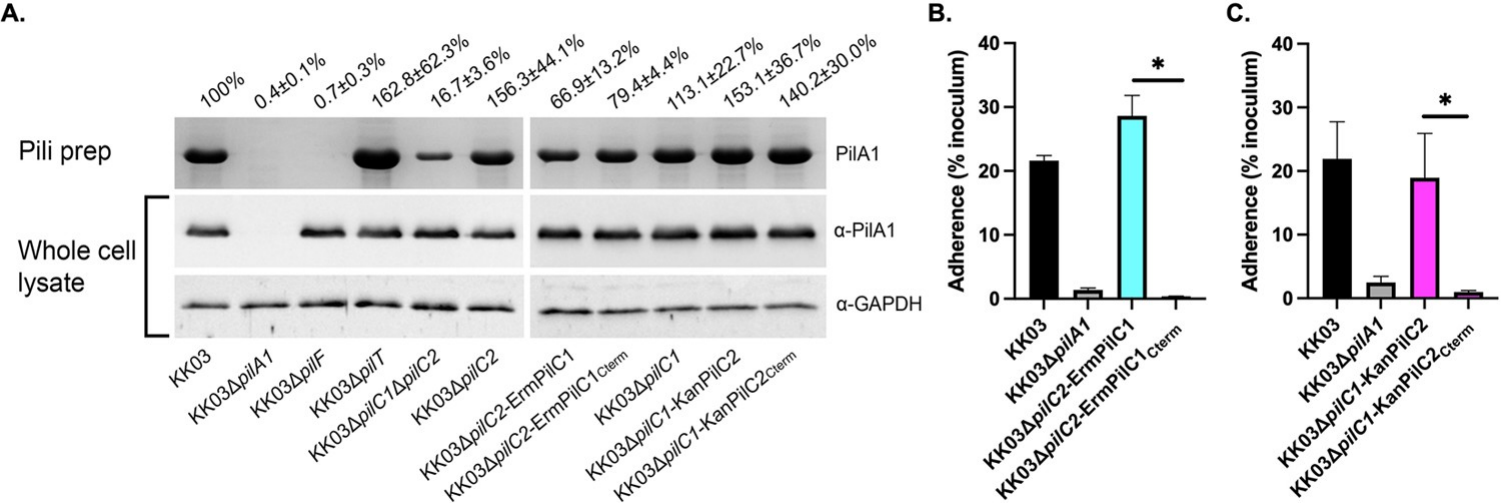
The PilC1 and PilC2 C-terminal domains promote production of pilus fibers that lack adhesive activity. (A) Sheared pili fractions or whole cell sonicates of strains KK03, KK03Δ*pilA1*, KK03Δ*pilF*, KK03Δ*pilT*, KK03Δ*pilC1*Δ*pilC2*, KK03Δ*pilC2*, KK03Δ*pilC2-*ErmPilC1, KK03Δ*pilC2-*ErmPilC1_Cterm_, KK03Δ*pilC1*, KK03Δ*pilC1-*KanPilC2, and KK03Δ*pilC1*-KanPilC2_Cterm_ were boiled and separated using SDS-PAGE. KK03Δ*pilF* lacks the PilF assembly ATPase and does not assemble PilA1 into pilus fibers. For pili preps, the PilA1 pilin monomer band was stained with Coomassie blue. For whole cell lysates, the PilA1 pilin monomer band was detected by Western blot analysis using polyclonal antiserum GP65 [52] to PilA1. GAPDH was detected by Western blot analysis to control for total protein using polyclonal antiserum CHP-GP22. The values above the gel and blot images are densitometry measurements of the PilA1 band from the sheared pilus preparations (pili prep) gel that were normalized to the α-GAPDH band intensities and are expressed as a percentage of the band intensity of the wild type strain KK03. The densitometry values are listed ± standard error of the mean from three biological replicates, and representative gel and blot images are shown. (B, C) Strains KK03, KK03Δ*pilA1*, KK03ErmΔ*pilC2-*PilC1, KK03Δ*pilC2-*ErmPilC1_Cterm_, KK03Δ*pilC1-*KanPilC2, and KK03Δ*pilC1*-KanPilC2_Cterm_ were added to monolayers of Chang epithelial cell of HeLa origin and evaluated for adherence. Percent adherence was calculated based on the ratio of recovered bacteria to the inoculum. Error bars represent standard error of the mean, n = 3. Cyan color denotes strains expressing either full-length PilC1 or the C-terminal region of PilC1 only. Magenta color denotes strains expressing either full-length PilC2 or the C-terminal region of PilC2 only. *indicates significance of P < 0.05 as determined by a one-way ANOVA using Bonferroni correction for multiple comparisons.

Previous work on the *P*. *aeruginosa* PilY1 protein established that the PilY1 C-terminal domain has a β-propeller fold and plays a critical role in *P*. *aeruginosa* type IV pilus assembly [26]. To localize the adhesive regions of PilC1 and PilC2, we generated deletion constructs lacking the N-terminal domains but containing intact C-terminal β-propeller folds (KK03Δ*pilC2*-ErmPilC1_Cterm_ and KK03Δ*pilC1*-KanPilC2_Cterm_) based on modeling with Phyre2 software [27] (S1 Fig). We hypothesized that the strains expressing only the C-terminal region of PilC1 or PilC2, in the absence of the other PilC, would still produce type IV pili but that these fibers would not be able to mediate adherence due to lack of the adhesive N-terminal region. Strains KK03Δ*pilC2*-ErmPilC1 and KK03Δ*pilC1*-KanPilC2, which contain an antibiotic marker upstream of the wild-type *pilC1* gene or downstream of the *pilC2* gene, respectively, were used as controls when evaluating the phenotypes of strains KK03Δ*pilC2*-ErmPilC1_Cterm_ and KK03Δ*pilC1*-KanPilC2_Cterm_. As shown in Fig 4A, piliation was reduced in strain KK03Δ*pilC2*-ErmPilC1 compared to the parent strain KK03Δ*pilC2*, indicating that the erythromycin resistance marker upstream of *pilC1* has a negative impact on pili levels. However, comparison of strains KK03Δ*pilC2*-ErmPilC1 and KK03Δ*pilC2*-ErmPilC1_Cterm_ revealed similar piliation levels, indicating that the PilC1_Cterm_ fragment is able to promote piliation at levels equivalent to full-length PilC1. Similarly, comparison of strain KK03Δ*pilC1*-KanPilC2 with strain KK03Δ*pilC1*-KanPilC2_Cterm_ revealed equivalent levels of piliation, indicating that the PilC2 C-terminal domain is also able to promote similar piliation levels as full-length PilC2. Despite the presence of pili, these strains were non-adherent in assays with cultured Chang cells of HeLa origin (Fig 4B and 4C), suggesting that the N-terminal domains of PilC1 and PilC2 are critical for adhesive activity. These results indicate that the C-terminal domains of PilC1 and PilC2 are sufficient for pilus assembly and suggest that the N-terminal domains of PilC1 and PilC2 harbor adhesive activity.

### The *K*. *kingae* PilC1 and PilC2 N-terminal domains bind to epithelial cell monolayers

**Fig 5:**
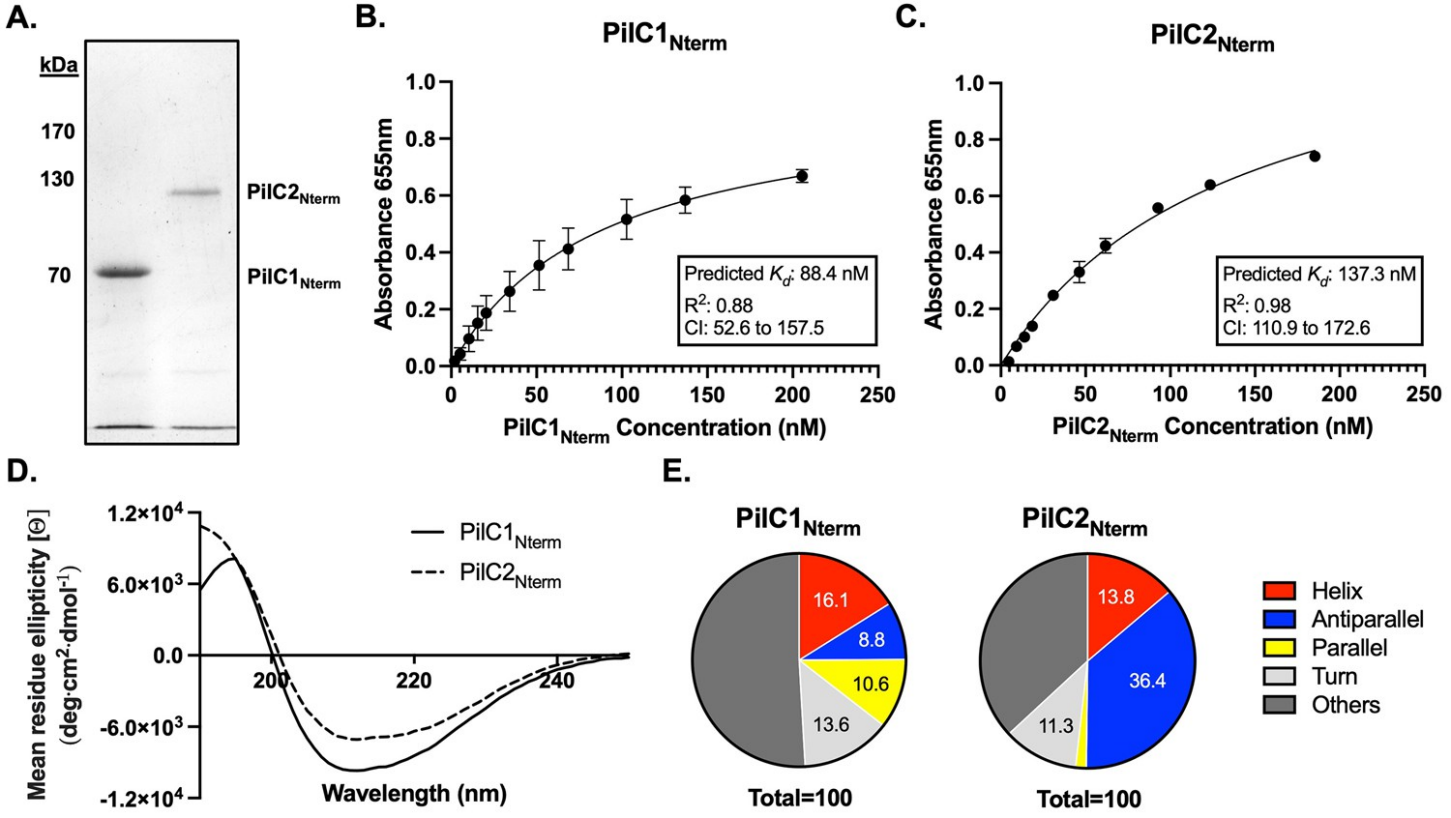
The PilC1 and PilC2 N-terminal domains bind to epithelial cell monolayers. (A) Recombinant PilC1 N-terminal domain (PilC1_Nterm_) and PilC2 N-terminal domain (PilC2_Nterm_) were purified and separated on a 7.5% SDS PAGE gel and stained with Coomassie blue. (B, C) Proteins were added to monolayers of Chang epithelial cells of HeLa origin at increasing concentrations in 50 mM Tris-HCl, pH 8.5. Adherence was detected by ELISA using polyclonal antiserum CHP-R1 for PilC1_Nterm_ (B) or GP103 for PilC2_Nterm_ (C) and a secondary anti-rat or anti-guinea pig antibody conjugated to HRP. Non-linear regressions were fit using the GraphPad Prism one site specific binding model fit to total data from 3 independent runs, and the *K*_*d*_ was calculated based on the regression. Error bars represent standard error of the mean, n = 3. (D, E) Circular dichroism was carried out on purified PilC1_Nterm_ and PilC2_Nterm_ (D) and the resultant spectra were used to predict protein secondary structures (E). The CD spectra are representative graphs of three independent analyses carried out on different batches of purified protein.

To obtain definitive evidence that the adhesive activity of PilC1 and PilC2 resides in the N-terminal domains of these proteins, we purified recombinant truncated 6X histidine-tagged PilC1 and PilC2 proteins corresponding to the N-terminal domains, lacking the predicted β-propeller region (Figs 5A and S1). As shown in Fig 5B, the PilC1 N-terminal domain exhibited saturable binding with a predicted *K*_*d*_ of 88.4 nM, comparable to the *K*_*d*_ of full-length PilC1 (74.4 nM). Similarly, as shown in Fig 5C, the PilC2 N-terminal domain displayed saturable binding with a predicted *K*_*d*_ of 137.3 nM, comparable to the *K*_*d*_ of full-length PilC2 (165.6 nM). Based on CD spectroscopy (Fig 5D and 5E), the PilC1 N-terminal domain was predicted to contain 16.1% helices, 8.8% antiparallel sheets, 10.6% parallel sheets, and 13.6% turns, while the PilC2 N-terminal domain was predicted to contain 13.8% helices, 36.4% antiparallel sheets, 11.3% turns, and less than 2% parallel sheets. These data demonstrate that the PilC1 and PilC2 N-terminal domains harbor adhesive activity and appear to have distinct structures.

### The PilC1 and PilC2 C-terminal domains are not sufficient for twitching motility but support transformation

**Fig 6:**
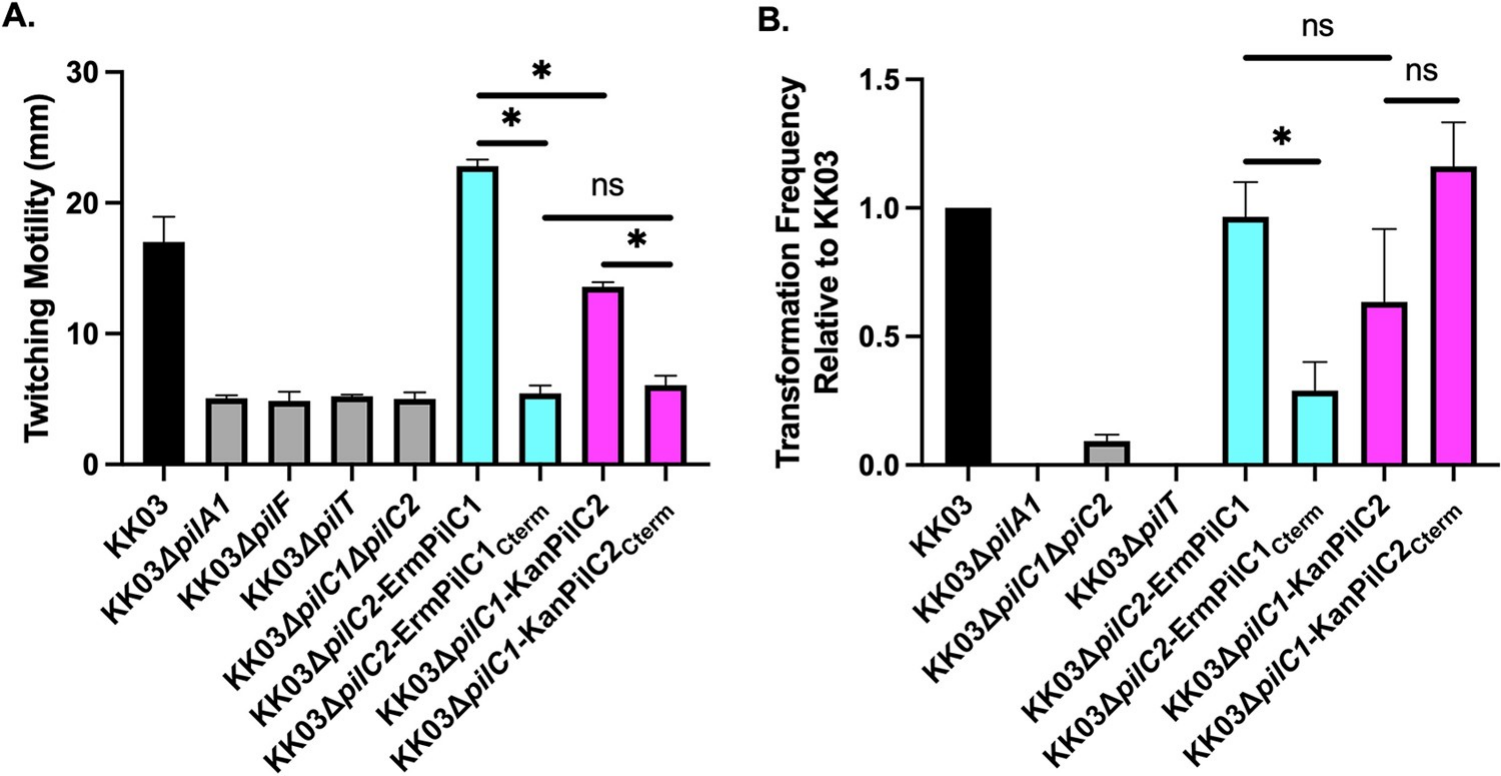
The PilC1 and PilC2 C-terminal domains are not sufficient for twitching motility but promote *K*. *kingae* natural transformation. (A) For strains KK03, KK03Δ*pilA1*, KK03Δ*pilF*, KK03Δ*pilT*, KK03Δ*pilC1*Δ*pilC2*, KK03Δ*pilC2*-ErmPilC1, KK03Δ*pilC2-*ErmPilC1_Cterm_, KK03Δ*pilC1-*KanPilC2, and KK03Δ*pilC1*-KanPilC2_Cterm_, the zone of twitching motility was stained with crystal violet and was quantified by measuring the diameter of bacterial spread. (B) Natural transformation was measured for strains KK03, KK03Δ*pilA1*, KK03Δ*pilC1*Δ*pilC2*, KK03Δ*pilT*, KK03Δ*pilC2-*ErmPilC1, KK03Δ*pilC2-*ErmPilC1_Cterm_, KK03Δ*pilC1-*KanPilC2, and KK03Δ*pilC1*-KanPilC2_Cterm_. Transformation frequency was calculated based on the ratio of recovered bacteria to inoculum. Error bars represent standard error of the mean, n = 3. Cyan color denotes strains expressing either full-length PilC1 or the C-terminal region of PilC1 only. Magenta color denotes strains expressing either full-length PilC2 or the C-terminal region of PilC2 only. *indicates significance of P < 0.05 as determined by a one-way ANOVA using Bonferroni correction for multiple comparisons. ns indicates no significance.

To begin to localize other PilC1- and PilC2-mediated functions, we examined twitching motility in strains KK03Δ*pilC2*-ErmPilC1_Cterm_ and KK03Δ*pilC1*-KanPilC2_Cterm_. As shown in Fig 6A, compared to strain KK03Δ*pilC2*-ErmPilC1, strain KK03Δ*pilC2*-ErmPilC1_Cterm_ was devoid of twitching motility, similar to the non-piliated Δ*pilA1* mutant. Compared to strain KK03Δ*pilC1*-KanPilC2, strain KK03Δ*pilC1*-KanPilC2_Cterm_ was also devoid of twitching motility. These results suggest that the C-terminal domains of PilC1 and PilC2 are not sufficient for promoting twitching motility, localizing this activity to either the N-terminal domains or to regions in both the N-terminal and the C-terminal domains.

Twitching motility requires sequential pilus extension, adhesion to a substrate, and retraction. To determine if the defect in twitching motility in KK03Δ*pilC2*-ErmPilC1_Cterm_ and KK03Δ*pilC1*-KanPilC2_Cterm_ was due to a defect in pilus retraction, we examined natural transformation in these strains, recognizing that natural transformation requires pilus retraction in other systems [28]. The WT strain exhibited transformation efficiencies ranging from 0.3% to 1.5%, normalized to 1.0 in Fig 6B. As expected, the non-piliated Δ*pilA1* mutant and the retraction-deficient Δ*pilT* mutant were completely non-transformable. Strain KK03Δ*pilC1*Δ*pilC2* was capable of low but measurable levels of transformation, while strains KK03Δ*pilC2*-ErmPilC1 and KK03Δ*pilC1*-KanPilC2 displayed wild type levels of transformation, suggesting that the presence of either PilC1 or PilC2 is sufficient for wild type transformation efficiency. While strain KK03Δ*pilC*1-KanPilC2_Cterm_ displayed wild type transformation efficiency, strain KK03Δ*pilC2*-ErmPilC1_Cterm_ exhibited a statistically significant decrease in transformation efficiency when compared to KK03Δ*pilC2*-ErmPilC1. This lower level of transformation was above the level observed for strain KK03Δ*pilC1*Δ*pilC2*, indicating that the PilC1 C-terminal domain promotes transformation and that full transformation efficiency requires additional sequence N-terminal to the C-terminal domain fragment. Taken together, these data indicate that the PilC1 and PilC2 C-terminal domains are sufficient for retraction, and thus the lack of twitching motility in KK03Δ*pilC2*-ErmPilC1_Cterm_ and KK03Δ*pilC1*-KanPilC2_Cterm_ is not a consequence of absent pilus retraction.

## Discussion

In this study we examined the role of the *K*. *kingae* PilC1 and PilC2 proteins in *K*. *kingae* adherence. Using purified full-length and truncated PilC1 and PilC2, we established that PilC1 and PilC2 are adhesins, with the adhesive activity located in the N-terminal domains of these proteins. Using deletion constructs, we found that PilC1-mediated and PilC2-mediated twitching motility requires the N-terminal domains. In contrast, our results indicate that the C-terminal domains of PilC1 and PilC2 are sufficient for promoting pilus assembly and natural transformation, although the PilC1 C-terminal domain is not sufficient for wild type transformation efficiency.

Earlier studies suggested that the *K*. *kingae* PilC1 and PilC2 proteins might be the adhesive components of the pilus, as expression of either the PilC1 or PilC2 protein is required for bacterial adherence to epithelial cells [7]. In this work, we found that deletion of the PilT retraction ATPase restored production of *K*. *kingae* surface fibers in the absence of a functional PilC protein but did not restore adherence. Furthermore, we observed that purified PilC1 and PilC2 were capable of binding to epithelial cell monolayers to saturating levels, providing strong evidence that these proteins are adhesins that interact with epithelial cells in a receptor-mediated process. Interestingly, the predicted *K*_*d*_ measurements of the *K*. *kingae* PilC1 and PilC2 N-terminal domains mimicked results with the full-length proteins, supporting the conclusion that full adhesive activity resides in the N-terminal domains [16].

Adhesins expressed by host-adapted bacteria commonly bind to extracellular matrix (ECM) components. Heiniger *et al*. showed that *P*. *aeruginosa* preferentially binds to the exposed basolateral cell surface of injured polarized epithelial cells in a PilY1-dependent manner [13]. As ECM proteins are located at the basolateral side of epithelial cells in the basement membrane and connective tissue, they are well positioned to serve as targets for bacterial adherence when there is damage to the epithelium. In this report, we demonstrate that *K*. *kingae* PilC1, but not PilC2, mediates bacterial adherence to ECM proteins. During the *K*. *kingae* pathogenic process, damage to the epithelial surface mediated by the *K*. *kingae* RTX toxin [21] or viral infection [29–32] may promote bacterial access to the basement membrane, where PilC1 may bind to ECM proteins, potentially facilitating invasive disease.

Our studies examining adherence to ECM proteins establish that the *K*. *kingae* PilC1 and PilC2 proteins have distinct binding specificities, consistent with the limited homology between PilC1 and PilC2 and the different global structures as assessed by CD spectroscopy. Interestingly, the *N*. *meningitidis* PilC1 and PilC2 proteins also have distinct binding specificities, despite the fact that these proteins are highly homologous. In particular, *N*. *meningitidis* PilC1 has been demonstrated to promote meningococcal adherence to HEC-1-B, ME180, and HUVEC cell lines, while PilC2 promotes adherence only to ME180 cells [33,34].

It is notable that *K*. *kingae* PilC1-mediated adherence to ECM proteins was increased in the absence of PilC2, similar to our earlier observation that PilC1-mediated twitching motility was increased in the absence of PilC2 [17]. We have established that PilC1 and PilC2 production is not affected by the presence or the absence of the other protein (S2 Fig), suggesting that changes in expression cannot account for the differences in the phenotypes observed. One possibility is that PilC2 obscures PilC1 along the pilus and interferes with PilC1 interaction with its receptor. Another possibility is that PilC1 localization along the pilus is affected when PilC2 is eliminated. A third possibility is that PilC1 and PilC2 interact with each other and modify the function of each other.

The PilC family of proteins share a predicted C-terminal β-propeller fold domain. This fold was first identified when the C-terminal region of the *P*. *aeruginosa* PilY1 protein was crystallized [26], revealing a calcium-binding motif that is present in the β-propeller region of multiple PilC proteins [16,17,26,35]. Orans *et al*. showed that prevention of calcium-binding resulted in a defect in surface piliation and twitching motility, while mimicking the charge of a bound calcium ion produced an abundance of non-functional surface pili [26], suggesting a role for this domain in pilus biogenesis and retraction. Porsch *et al*. demonstrated that the *K*. *kingae* PilC1 and PilC2 calcium-binding motifs promoted twitching motility [17]. In this study, we observed that the C-terminal domain of *K*. *kingae* PilC1 and PilC2 was sufficient for promoting surface piliation, consistent with a role for the C-terminal domain in supporting pilus dynamics.

Studies in gram-negative bacteria have shown that T4P components and PilC proteins promote natural competence for transformation [28,36–38]. Because the expression of at least one PilC protein is required for surface piliation, ascertaining whether the proteins promote DNA uptake by promoting piliation or through another mechanism has been a challenge. KK03Δ*pilC2*-ErmPilC1_Cterm_ displays reduced transformation efficiency compared to strain KK03Δ*pilC2*-ErmPilC1 despite expressing similar levels of pili (Fig 4A), suggesting that factors beyond piliation level influence transformation efficiency. Other possible mechanisms of PilC protein involvement include direct binding of DNA or influencing other known competence proteins such as the pilus-associated ComP, which binds to DNA [39,40], or the periplasmic ComE or ComA, which function to promote DNA uptake and translocation [41–47]. As PilC1 and PilC2 localize to surface pili (S2 Fig), it is feasible that they may interact with ComE or ComA in the periplasm to modulate DNA translocation, or ComP along the pilus fiber to modulate DNA binding. The transformation defect observed in KK03Δ*pilC2*-ErmPilC1_Cterm_ but not KK03Δ*pilC1*-KanPilC2_Cterm_ raises the possibility that PilC1 and PilC2 have different functions during transformation. The PilC1 N-terminal region may function in combination with the C-terminal region to promote transformation, while the PilC2 C-terminal region may function alone to promote transformation using a distinct mechanism.

Twitching motility is believed to be carried out by sequential T4P extension, adhesion to a substrate, and PilT-mediated retraction of the adhering pilus fibers, which exert a pulling force allowing the bacteria to move along a surface [28,48–51]. In PilC-containing bacteria, at least one functional PilC protein is required for a twitching phenotype [10,17,26]; however, it remains to be determined if the PilC proteins promote twitching motility by promoting adherence, by regulating the pilus extension/retraction dynamic, or both. Strains KK03Δ*pilC2*-ErmPilC1_Cterm_ and KK03Δ*pilC1*-KanPilC2_Cterm_ produce retractile pilus fibers, as evidenced by their natural transformation. Despite being retractile, the present fibers are unable to promote bacterial adherence to epithelial cells. For this reason, the defect observed in twitching motility is likely due to a defect in adherence, rather than a defect in pilus retraction.

Our data demonstrate that *K*. *kingae* PilC1 and PilC2 differentially promote *K*. *kingae* adherence using the N-terminal domains to directly interact with epithelial cells and the C-terminal domains to promote surface piliation. Future studies should elucidate the specific adhesive motifs in PilC1 and PilC2 and should address whether PilC1 and PilC2 interact with each other to modulate type IV pilus-mediated phenotypes.

## Methods

### Generation of *K*. *kingae* mutants

*K*. *kingae* gene disruptions and mutations were generated as described previously [7,17,52]. Plasmid-based disruption and truncation constructs were generated in *E*. *coli*, linearized, and introduced into *K*. *kingae* using natural transformation of linearized plasmid DNA followed by selection for mutants on chocolate agar plates with the appropriate antibiotic. Mutations were confirmed by genomic DNA preparation of putative mutant strains followed by PCR amplification and evaluation by Sanger sequencing.

To generate the unmarked KK03Δ*pilC2* mutant, the plasmid pUC19/Δ*pilC2* [17] lacking a resistance cassette was transformed into the kanamycin-marked strain KK03Δ*pilC2* using the spot transformation technique [53], and patch plating was used to screen for the loss of kanamycin resistance. To generate strain KK03Δ*pilC1*Δ*pilC2*, linearized pUC19/Δ*pilC1* [7] was introduced into KK03Δ*pilC2* and transformants were recovered by selection on chocolate agar with 2 μg/ml tetracycline. To generate strain KK03Δ*pilC2*Δ*pilT*, linearized pUC19/Δ*pilT* [17] DNA was introduced into strain KK03Δ*pilC2* and transformants were recovered by selection on chocolate agar with 1 μg/ml erythromycin. To generate strain KK03Δ*pilC1*Δ*pilT*, linearized pUC19/Δ*pilT* [17] DNA was introduced into strain KK03Δ*pilC1* [17], as described above. To generate strain KK03Δ*pilC1*Δ*pilC2*Δ*pilT*, linearized pUC19/Δ*pilT* [17] DNA was introduced into strain KK03Δ*pilC1*Δ*pilC2* as described above. For pUC19/Δ*pilA1*:*tet*, the *aphA3* kanamycin resistance cassette in pUC19/Δ*pilA1*:*kan* [7] was excised as a MluI-fragment and replaced with a *tetM* tetracycline resistance cassette. For strain KK03Δ*pilA1*, linearized plasmid pUC19/Δ*pilA1*:tet DNA was introduced into KK03 and transformants were recovered by selection on chocolate agar with 2 μg/ml tetracycline.

To generate strain KK03Δ*pilC2*-ErmPilC1_Cterm_, site-directed mutagenesis using primers C1ΔNterm_mut_sense and C1ΔNterm_mut_anti (see Table 2 for the primer sequences used in this study) and the QuikChange II XL Site-directed mutagenesis kit (Agilent, Santa Clara, CA) was used to delete sequence encoding amino acids 32–438, using plasmid pUC19/Erm*pilC1* [17] as the template. The C-terminal domains were designated as the region of the proteins encompassing the predicted β-propeller fold based on modeling with Phyre2 software [27], and the N-terminal regions were defined as the polypeptide excluding the signal sequence and the predicted β-propeller fold. The resulting construct, pUC19/Erm*pilC1*ΔNterm, which contains an erythromycin resistance marker upstream of the *pilC1* promoter region and the PilC1 signal sequence fused in-frame to the C-terminal domain, was linearized and transformed into strain KK03Δ*pilC2*, generating strain KK03Δ*pilC2*-ErmPilC1_Cterm_. To generate strain KK03Δ*pilC1*-KanPilC2_Cterm_, Gibson assembly was employed using DNA fragments amplified with Q5 Hi-Fidelity Master Mix with primer pairs *pilC2*up_F and *pilC2*up_R (Table 2) (using KK03 genomic DNA as template) and *pilC2*downKan_F and *pilC2*downKan_R (using KK03Δ*pilC1*-KanPilC2 [17] genomic DNA as the template) and EcoRI-digested pUC19. The resulting plasmid, pUC19/*pilC2*ΔNtermKan, which contains sequence encoding the PilC2 signal sequence fused in-frame to sequence encoding the C-terminal domain and a kanamycin-resistance marker downstream of the *pilC2* ORF, was linearized and transformed into strain KK03Δ*pilC1*, generating strain KK03Δ*pilC1*-KanPilC2_Cterm_. A schematic representation of the *pilC1* and *pilC2* loci in the panel of mutants used in this study is shown in S3 Fig.

**Table 2:** Primers used in this study.

Primer	Sequence (5’ → 3’)
GibCytoC1F	TTTAAGAAGGAGATATACATATGCATCATCACCATCACCAC
GibCytoC1R	CAGTGGTGGTGGTGGTGGTGCTCGAGTTAGAAAATCTCACGCCAAG
GibCytoC2F	CTTTAAGAAGGAGATATACATATGCATCATCACCATCACCACAACAATACCCCTTTTTCTGATTC
GibCytoC2R	AGTGGTGGTGGTGGTGGTGCTTAGAAAATCTCGCGCCAAG
CNtermF	CTTTAAGAAGGAGATATACATATGCATCATCACCATCACCAC
C1NtermR	AGTGGTGGTGGTGGTGGTGCTTATTGCGCAATGCCATTTTTAC
C2NtermR	AGTGGTGGTGGTGGTGGTGCTTAGCTAATATTGGCAGATG
C1ΔNterm_mut_sense	GCGACAATGGCGGCTGATGTACAAAAAGCGTACTATT
C1ΔNterm_mut_anti	AATAGTACGCTTTTTGTACATCAGCCGCCATTGTCGC
*pilC2*up_F	TTGTAAAACGACGGCCAGTGCACAACAGAGTAACACCATG
*pilC2*up_R	TGCCATCTTCGTTTTCTGCTTGTGCATAAAAAG
*pilC2*downKan_F	AGCAGAAAACGAAGATGGCAGAAAATTCCG
*pilC2*downKan_R	ATCCCCGGGTACCGAGCTCGGGTAATCCAGCACGAGATTTG
pBAD*pilC1_*F	CGTTTTTTTGGGCTAGCGGTGAGTAGTATGAGTCTTAAATATAAAAAATATAATTG
pBAD*pilC1_*R	CGGGTACCGAGCTCGATTTTTGTGAATACAGGTTCTG
*gapdh*_F	ACGTGTCGACATGAGCGTTAAAGTAGCCATTAAC
*gapdh*_R	AGCTGAATTCATAATAATCAAAGCAGCCTGCAC

For the transformation efficiency experiments, the plasmid pTrc99a/*knh*:*tetM* [8] was modified by excising the *tetM* cassette as a ClaI fragment and inserting the *aphA3* kanamycin resistance cassette or the *ermC* erythromycin resistance cassette, generating pTrc99a/*knh*:*aphA3* and pTrc99a/*knh*:*ermC*, respectively.

### Eukaryotic cell lines

Chang epithelial cells (Wong-Kilbourne derivative [D] of Chang conjunctiva, HeLa origin; ATCC CCL-20.2) were grown at 37°C, 5% CO_2_ in Modified Eagles’ Medium (MEM) supplemented with 10% fetal bovine serum (FBS) and non-essential amino acids diluted 1:100, as described previously [21].

### Generation of recombinant PilC1 and PilC2 constructs

Sequences corresponding to the ORFs of *pilC1* and *pilC2* lacking sequence encoding the predicted signal sequences were amplified from genomic DNA from either KK03 or KK03 producing 6X histidine-tagged PilC1 using Q5 High-Fidelity Master Mix (New England Biolabs, Ipswich, MA) with the primers GibCytoC1F and GibCytoC1R for PilC1 and GibCytoC2F and GibCytoC2R for PilC2. The signal sequence cleavage sites were predicted using SignalP [54,55]. The 6X histidine tag was placed at the N-terminus of the protein immediately following the predicted signal peptide cleavage site. The recombinant PilC1 full-length protein encompassed amino acids Asp_35_ –Phe_1359_, and the recombinant PilC2 full-length protein encompassed amino acids Asn_37_ –Phe_1502_. Gibson assembly was used to introduce sequence encoding 6X histidine-tagged PilC1 and PilC2 into the vector pET22b, generating pET22b-PilC1 and pET22b-PilC2, respectively. For PilC2 an N-terminal 6X histidine tag was added using primers GibCytoC2F and GibCytoC2R. The assembly reaction was carried out using the Gibson Assembly Master Mix (New England Biolabs) according to the manufacturer’s instructions, and the reaction was transformed into electrocompetent *E*. *coli* DH5α followed by the selection for transformants on LB agar with 100 μg/ml ampicillin. The plasmids were purified, and the constructs were confirmed using Sanger sequencing. The N-terminal regions of PilC1 and PilC2 were amplified from pET22b-PilC1 and pET22b-PilC2 using Q5 High-fidelity Master Mix with primers CNtermF and C1NtermR for PilC1 and CNtermF and C2NtermR for PilC2. The C-terminal domains were designated as the region of the proteins encompassing the predicted β-propeller fold based on modeling with Phyre2 software [27], and the N-terminal regions were defined as the polypeptide excluding the signal sequence and the predicted β-propeller fold. The amplified PilC1 N-terminal region encompassed amino acids Asp_35_ –Gln_672_, while the PilC2 N-terminal region encompassed amino acids Asn_37_ –Ser_1022_. Gibson assembly was used to introduce sequence encoding the 6X histidine-tagged PilC1 N-terminal region and the 6X histidine-tagged PilC2 N-terminal region into pET22b, generating pET22b-PilC1_Nterm_ and pET22b-PilC2_Nterm_, which were purified and confirmed using Sanger sequencing. The confirmed constructs were introduced into electrocompetent *E*. *coli* BL21 followed by selection for transformants on LB agar with 100 μg/ml ampicillin.

### PilC1 and PilC2 protein purification

Overnight cultures of *E*. *coli* BL21 with either pET22b-PilC1, pET22b-PilC2, pET22b-PilC1_Nterm_, or pET22b-PilC2_Nterm_ were back-diluted 1:200 in LB broth with 100 μg/ml ampicillin. After reaching an OD_600_ of 0.4, gene expression was induced for 3 hours at 30°C using 0.04 mM isopropyl β-d-1-thiogalactopyranoside (IPTG). Subsequently, the cultures were centrifuged at 6700 x g for 20 minutes, and the supernatant was discarded. The cell pellets were resuspended in 50 mM tris-HCl, 5 mM EDTA, and 10 mM NaCl at pH 8.0 at a ratio of 3 ml buffer to 1 gram of cells. AEBSF was added to a final concentration of 1 mM. For each 1 gram of cells, 0.8 mg of lysozyme was added. The solution was mixed and placed in a 37°C water bath until viscous. The solution was sonicated 3 x at 25% amplitude for 30 seconds using a QSonica Q500 sonicator to shear DNA. The solution was centrifuged at 39,191 x g for 30 minutes, and the supernatant was discarded. The resulting pellets were resuspended in 20 mM Na_2_HPO_4_, 20 mM NaCl, 5 mM EDTA, and 25% sucrose at pH 7.2 at a ratio of 3 ml buffer to 1 gram of material. AEBSF was again added to a final concentration of 1 mM. Triton X-100 was added to a final concentration of 1%. The solution was mixed and centrifuged at 48,384 x g for 20 minutes, and the supernatant was discarded. The pellets were solubilized using 50 mM tris-HCl, 40 mM imidazole, 8 M urea, and 1mM β-mercaptoethanol at pH 8.0 (solubilization buffer) for several hours at 37°C, adding more buffer until no more pellet would solubilize. The solubilized pellet was centrifuged at 48,384 x g for 20 minutes. The remaining supernatant was filtered using vacuum filtration through a 0.22 μM membrane, and the 6X histidine-tagged PilC1, PilC2, PilC1_Nterm_, or PilC2_Nterm_ was purified from the supernatant using affinity chromatography over an Ni-NTA agarose column. The 2 ml column bed volume was equilibrated with the solubilization buffer, and the supernatants were added to the column and incubated at room temperature on a rotator for 3 hours to facilitate protein-binding. The column was washed with 20 ml of solubilization buffer. Proteins were eluted with 50 mM tris-HCl, 500 mM imidazole, 8 M urea, and 1 mM β-mercaptoethanol at pH 8.0. The samples were concentrated over a 100,000 Da molecular weight cutoff filter for PilC1 and PilC2 or a 50,000 Da molecular weight cutoff filter for PilC1_Nterm_ and PilC2_Nterm_. The samples were then dialyzed into 1 L 50 mM tris-HCl at pH 8.5 at 4°C for 24 hours. The buffer was removed and replaced with 1 L fresh buffer, and samples were dialyzed at 4°C for an additional 24 hours. The protein samples were stored at 4°C in 50 mM Tris-HCl at pH 8.5.

### Generation of polyclonal antisera

To generate a guinea pig antiserum to PilC1, the sequence encoding PilC1 minus the predicted signal peptide was amplified from KK03 genomic DNA with primers pBAD*pilC1_*F and pBAD*pilC1*_R and was cloned into KpnI-digested pBAD18 using Gibson assembly. The resulting plasmid pBAD*-pilC1* was transformed into electrocompetent DH5α, and gene expression was induced with 0.2% arabinose. The bacterial pellet was harvested and sonicated. After centrifugation, the insoluble fraction containing recombinant PilC1 was separated on a 7.5% SDS-PAGE gel and stained with Coomassie blue, and the PilC1 band was excised and sent to Cocalico Biologicals to immunize a guinea pig (CHP-GP7) according to their standard antiserum generation protocol. To generate a rat antiserum to PilC1, 6XHisPilC1 was used to immunize a rat (CHP-R1) at Cocalico Biologicals according to their standard antiserum generation protocol. To generate an antiserum to PilC2, a PilC2 fragment corresponding to amino acids 868–1502, generated as previously described [17], was used to immunize a guinea pig (GP103) at Cocalico Biologicals according to their standard antiserum generation protocol. To generate a rabbit polyclonal antiserum to the PilC1 N-terminal region, recombinant PilC1_Nterm_ was used to immunize a rabbit (Rab128) at Cocalico Biologicals according to their standard antiserum generation protocol.

To generate an antiserum to the *K*. *kingae* glyceraldehyde phosphate dehydrogenase (GAPDH) protein, the full *gapdh* gene sequence (minus the start codon) was amplified using Q5 High-Fidelity Master Mix with primers *gapdh*_F and *gapdh*_R using KK03 genomic DNA as template. The resulting amplicon was digested with EcoRI and BamHI and ligated into EcoRI/BamHI-digested pHAT10, generating plasmid pHAT10-*gapdh*, encoding N-terminal histidine affinity tag (HAT)-tagged GAPDH. The plasmid was transformed into *E*. *coli* BL21(DE3), and expression of the recombinant *gapdh* gene was induced with 0.4 mM IPTG for 3 hours at 30°C. The resulting bacterial pellet was suspended in binding/wash buffer (20 mM sodium phosphate pH 7.4, 500 mM NaCl, 40 mM imidazole), sonicated, clarified via centrifugation at 20,000 x g for 20 min, and applied to a pre-equilibrated 5 ml HisTrap column (Cytiva, Marlborough, MA), using an AKTA Protein Purifier 10. After washing with 10 column volumes of binding/wash buffer, the bound protein was eluted with 20 mM sodium phosphate pH 7.4, 500 mM NaCl, 500 mM imidazole. The fractions containing the fusion protein were pooled, buffer exchanged and concentrated in 20 mM sodium phosphate pH 7.4, 500 mM NaCl using a 10,000 Da molecular weight cutoff filter, and then frozen at -80°C. The purified fusion protein was then sent to Cocalico Biologicals for injection into a guinea pig (CHP-GP22) using their standard antiserum generation protocol. Reactivity of all antisera was assessed using Western blot analysis.

### Cell-based protein-binding assays

Chang epithelial cells of HeLa origin were seeded into 96 well tissue culture-treated plates at a density of 3.24 x 10^4^ cells per well, and the plates were incubated overnight for ~18 hours at 37°C, 5% CO_2_. The cells were then fixed with 2% glutaraldehyde in sodium phosphate buffer and washed 3x with Tris-buffered saline (TBS). The monolayers were blocked using 2% dry milk powder in phosphate-buffered saline (PBS) for 1.5 hours at 37°C_._ Protein dilutions ranging from 1.85 nM to 205.55 nM were prepared in 50 mM Tris-HCl, pH 8.5. The blocking buffer was removed, and the diluted proteins were added to either tissue culture treated plates only or tissue culture treated plates coated with epithelial cell monolayers. Protein-loaded plates were incubated at 37°C for 3 hours before being washed 4 x with PBS. Polyclonal antisera diluted 1:500 in 2% dry milk powder/PBS was added to the plates, which were incubated at 37°C for 45 minutes and then washed 4 x with PBS. A secondary antibody conjugated to horseradish peroxidase diluted 1:2000 in 2% dry milk powder/PBS was added to the plates, and plates were incubated at 37°C for 45 minutes before being washed 4 x with PBS. Peroxidase substrate (3, 3’, 5, 5’–Tetramethylbenzidine) was added to the plates, and the color change was measured at 655 nm after 11 minutes of development using a multimode plate reader. Non-specific protein adherence to the plate and antibody adherence to epithelial cells were subtracted from the total protein adherence detected to epithelial cell monolayers to generate specific binding data.

### Circular dichroism

PilC1, PilC2, PilC1_Nterm_, and PilC2_Nterm_ proteins were diluted to a final concentration of 50 μg/ml in 16 mM tris-HCl, pH 8.5 and analyzed using a Jasco J-810 spectropolarimeter, using a 0.1 cm cuvette at a wavelength range of 250–190 nm. The spectropolarimeter was blanked using 16 mM Tris-HCl, pH 8.5 prior to taking measurements. The parameters were set to 1 nm data pitch, standard sensitivity, 1 s DIT, 1 nm bandwidth, immediate start mode, 20 nm/min scan speed, and 6 total accumulations. Spectra were converted to mean residue ellipticity to account for differences in the size of the proteins, and protein secondary structures were predicted using BeStSel [56,57].

### Quantitative bacterial adherence assays

Quantitative adherence assays were performed as described previously [7,8,17,52]. For determining adherence levels, Chang epithelial cells of HeLa origin were seeded into 24-well tissue culture treated plates and incubated overnight at 37°C, 5% CO_2_. The cells were then fixed with 2% glutaraldehyde in 0.2 M sodium phosphate buffer pH 7.4 and washed 3 x with TBS. For determining adherence to extracellular matrix (ECM), BioCoat plates coated with either collagen I, collagen IV, fibronectin, or laminin were purchased from Corning (Corning, NY). The ECM plates were equilibrated at room temperature for one hour prior to inoculation. The bacteria were cultured on chocolate agar plates at 37°C, 5% CO_2_ for 18–20 hours, swabbed from the plate, and resuspended in brain heart infusion media (BHI) to an OD_600_ of 0.8. A volume of 10 μl of resuspended bacteria was added to 300 μl of prewarmed 37°C MEM in 24-well tissue culture treated plates coated with either epithelial cell monolayers or ECM. The plates were incubated at 37°C, 5% CO_2_ for 25 minutes and were then washed 4 x with PBS to remove unbound bacteria. A volume of 100 μl 0.05% trypsin-EDTA was added to the plates, followed by incubation at 37°C, 5% CO_2_ for 20 minutes to facilitate bacterial recovery. The recovered bacteria were diluted and plated onto chocolate agar, and percent adherence was calculated based on the ratio of recovered bacteria to the inoculum.

### Pilus preparations

Pilus preparations were performed using a large-scale method modified from the small-scale method described previously [17]. Bacteria were grown at 37°C, 5% CO_2_ for 20 hours on chocolate agar plates, swabbed from the plate, and resuspended in 12 ml PBS to an OD_600_ of 0.8. Samples were vortexed at full speed for 1 min and centrifuged at 4,000 x g for 30 min to pellet bacteria. A total of 10 ml of the bacteria-free supernatant was subjected to 20% ammonium sulfate precipitation on ice for 2 hours. Precipitated pili were collected via centrifugation at 20,000 x g for 20 min and resuspended in 1x SDS-PAGE loading buffer. Pilus preparations were separated on 15% SDS-PAGE gels, stained with Coomassie blue, and imaged with a Syngene G:Box system.

### Densitometry analysis

To quantitate piliation levels, the PilA1 major pilin subunit band densities of Coomassie blue-stained pilus preparations and the Western blot GAPDH band densities of whole cell lysates (from the same bacterial pellet from which the pili were sheared for the pilus prepartions) were measured using ImageJ software [58,59]. The PilA1 band densities were divided by their matched GAPDH loading control band densities for normalization. The normalized values were then divided by the wild type KK03 normalized value to set wild type to 100% and compare all of the other strains to the wild type level. This analysis was completed for three independent biological replicates, and the average relative normalized PilA1 levels are presented ± standard error of the mean.

### Transformation efficiency assays

Strains were suspended to an OD_600_ of 0.8 in BHI broth, and 250 μl of the bacterial suspension was added to wells of a 24-well plate. A total of 1.0 μg of plasmid DNA containing either a kanamycin or erythromycin resistant cassette in place of the *knh* gene, using a plasmid backbone previously used to generate a *knh* deletion via allelic exchange [8], was added to the bacterial suspension. The transformation mixture was left at room temperature for 30 minutes, followed by the addition of 250 μl of BHI/20% lysed horse blood. The mixture was incubated at 37°C, 5% CO_2_ for 2.5 hours to allow for bacterial recovery before plating on chocolate agar containing 50 μg/ml kanamycin or 1 μg/ml erythromycin. The plates were incubated at 37°C, 5% CO_2_ overnight, and transformant colony forming units (cfu) were then enumerated. Transformation efficiency was calculated based on the ratio of kanamycin-resistant or erythromycin-resistant transformed cfu relative to the inoculum cfu.

### Twitching motility assays

Twitching motility assays were performed as described previously [17]. The strains were suspended to an OD_600_ of 0.8 in BHI. A 1 μl volume of the bacterial suspension was stab inoculated into the center of a chocolate agar motility plate (chocolate agar with 1% agar) to the plate-agar interface using a pipette tip. Plates were incubated at 37°C, 5% CO_2_ for three days. After three days the chocolate agar was removed, and the zone of bacterial spread at the plate-agar interface was stained with 0.1% crystal violet. The diameter of the crystal violet-stained bacterial spread was measured in millimeters.

## Supporting information

S1 Fig Schematic of full and truncated PilC1 and PilC2 proteins used in this study. PilC1, PilC1 C-terminal region (PilC1_Cterm_), PilC2, and PilC2 C-terminal region (PilC2_Cterm_) proteins expressed in *K*. *kingae* are shown with the included amino acids. Dashed boxes represent deleted regions of the protein. Black lines represent a fusion of the signal sequence with the C-terminal region. Black color represents the predicted signal sequence. The blue color represents the predicted PilC β- propeller domain. Recombinant PilC1 and PilC2 N-terminal proteins, expressed in *E*. *coli*, lack the signal sequence and are depicted as PilC1_Nterm_ and PilC2_Nterm_ in the diagram.
(TIF)

S2 Fig PilC1 and PilC2 localize to surface pili. Sheared pili fractions of strains KK03, KK03Δ*pilA1*, KK03Δ*pilC1*, KK03Δ*pilC2*, KK03Δ*pilC1*Δ*pilC2*, and KK03Δ*pilC1*Δ*pilC2*Δ*pilT* were boiled and separated using SDS-PAGE. PilC1 was detected by Western blot analysis using polyclonal antiserum Rab128 to PilC1_Nterm_, PilC2 was detected by Western blot analysis using polyclonal antiserum GP103 to PilC2, and GAPDH was detected by Western blot analysis using polyclonal antiserum GP22 to GAPDH. The PilA1 pilin monomer band was stained with Coomassie blue.
(TIF)

S3 Fig Cartoon representation of the *pilC1* and *pilC2* loci and *pilC1* and *pilC2* mutants used in this study. Strain KK03 (WT) produces PilC1 and PilC2, which are encoded by the *pilC1* and *pilC2* genes. Strain KK03Δ*pilC1*Δ*pilC2* contains a tetracycline resistance cassette in place of *pilC1* and an unmarked deletion of *pilC2* and does not produce PilC1 or PilC2. Strain KK03Δ*pilC2* contains an unmarked deletion of *pilC2* and produces full-length PilC1. Strains KK03Δ*pilC2-*ErmPilC1 and KK03Δ*pilC2-*ErmPilC1_Cterm_ contain an unmarked deletion of *pilC2* and an erythromycin resistance cassette upstream of *pilC1* and produce full-length PilC1 and the C-terminal domain of PilC1, respectively. Strain KK03Δ*pilC1* contains a tetracycline resistance cassette in place of *pilC1* and produces full-length PilC2. Strains KK03Δ*pilC1-*KanPilC2 and KK03Δ*pilC1-*KanPilC2_Cterm_ contain a tetracycline resistance cassette in place of *pilC1* and a kanamycin resistance cassette downstream of *pilC2* and produce full-length PilC2 and the C-terminal domain of PilC2, respectively. SS represents the predicted signal sequence. Cyan color denotes strains producing either full-length PilC1 or the C-terminal region of PilC1; magenta color denotes strains producing either full-length PilC2 or the C-terminal region of PilC2. The dashed lines indicate a deletion. Tet^R^, Kan^R^, and Erm^R^ indicate tetracycline, kanamycin, and erythromycin resistance cassettes, respectively.
(TIF)
