# Supplementary figures and images for: Kingella kingae PilC1 and PilC2 are adhesive multifunctional proteins that promote bacterial adherence, twitching motility, DNA transformation, and pilus biogenesis
Source: PLoS Pathog. 2022 Mar 30;18(3):e1010440. doi: 10.1371/journal.ppat.1010440 (PMC9000118; doi:10.1371/journal.ppat.1010440)

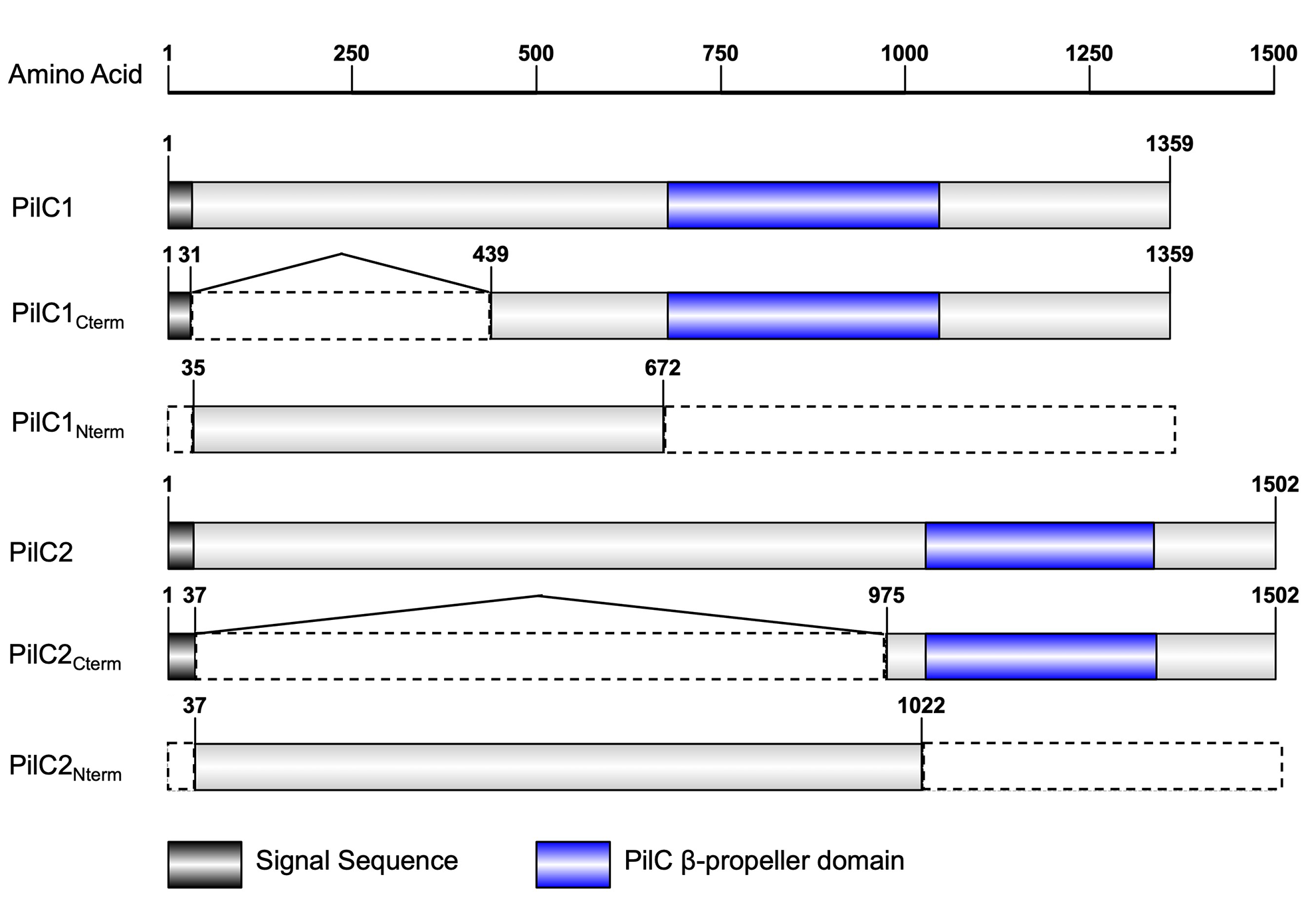

Supplement: S1 Fig — PilC1, PilC1 C-terminal region (PilC1Cterm), PilC2, and PilC2 C-terminal region (PilC2Cterm) proteins expressed in K. kingae are shown with the included amino acids. Dashed boxes represent deleted regions of the protein. Black lines represent a fusion of the signal sequence with the C-terminal region. Black color represents the predicted signal sequence. The blue color represents the predicted PilC β- propeller domain. Recombinant PilC1 and PilC2 N-terminal proteins, expressed in E. coli, lack the signal sequence and are depicted as PilC1Nterm and PilC2Nterm in the diagram. (TIF) [file ppat.1010440.s001.tif]

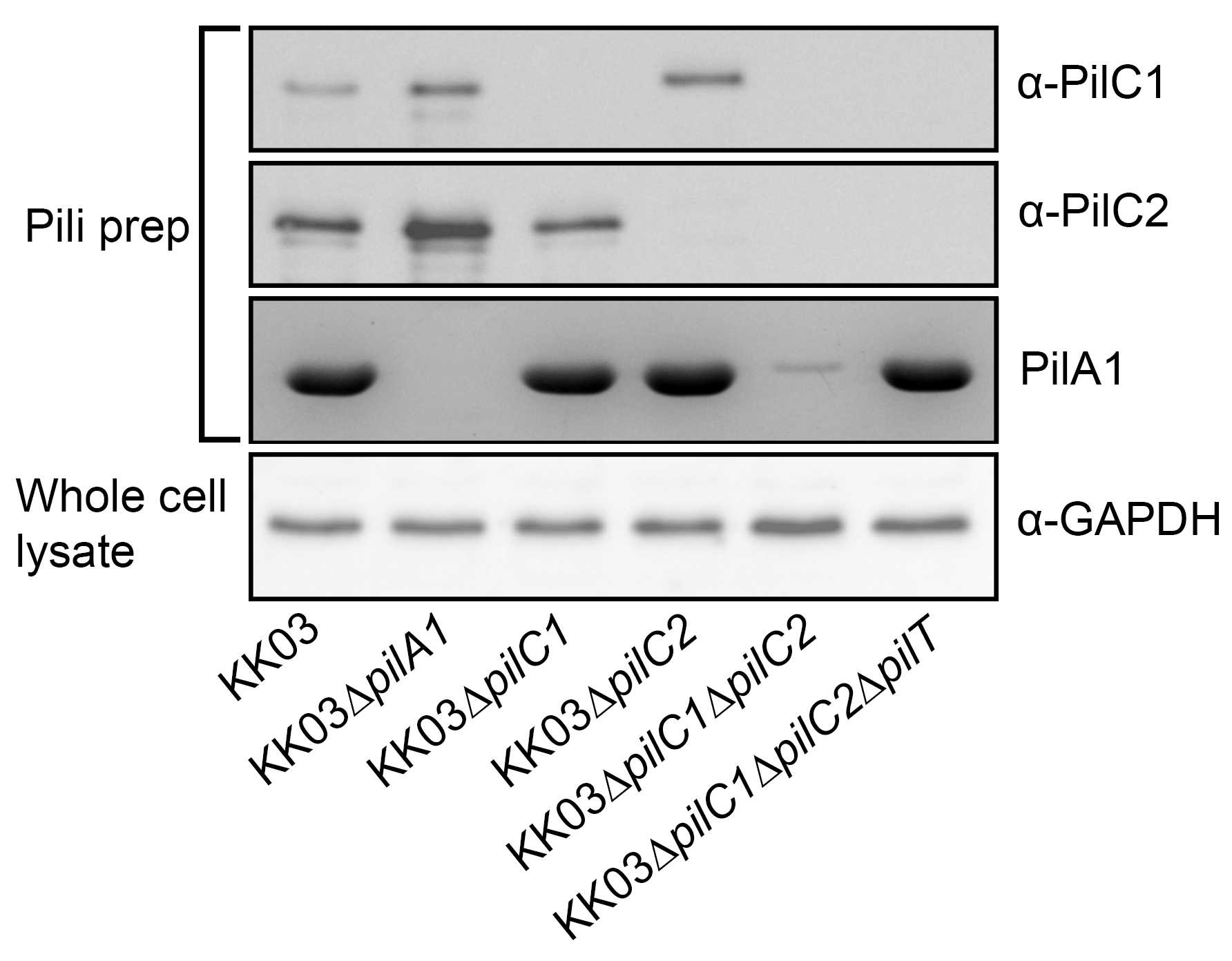

Supplement: S2 Fig — Sheared pili fractions of strains KK03, KK03ΔpilA1, KK03ΔpilC1, KK03ΔpilC2, KK03ΔpilC1ΔpilC2, and KK03ΔpilC1ΔpilC2ΔpilT were boiled and separated using SDS-PAGE. PilC1 was detected by Western blot analysis using polyclonal antiserum Rab128 to PilC1Nterm, PilC2 was detected by Western blot analysis using polyclonal antiserum GP103 to PilC2, and GAPDH was detected by Western blot analysis using polyclonal antiserum GP22 to GAPDH. The PilA1 pilin monomer band was stained with Coomassie blue. (TIF) [file ppat.1010440.s002.tif]

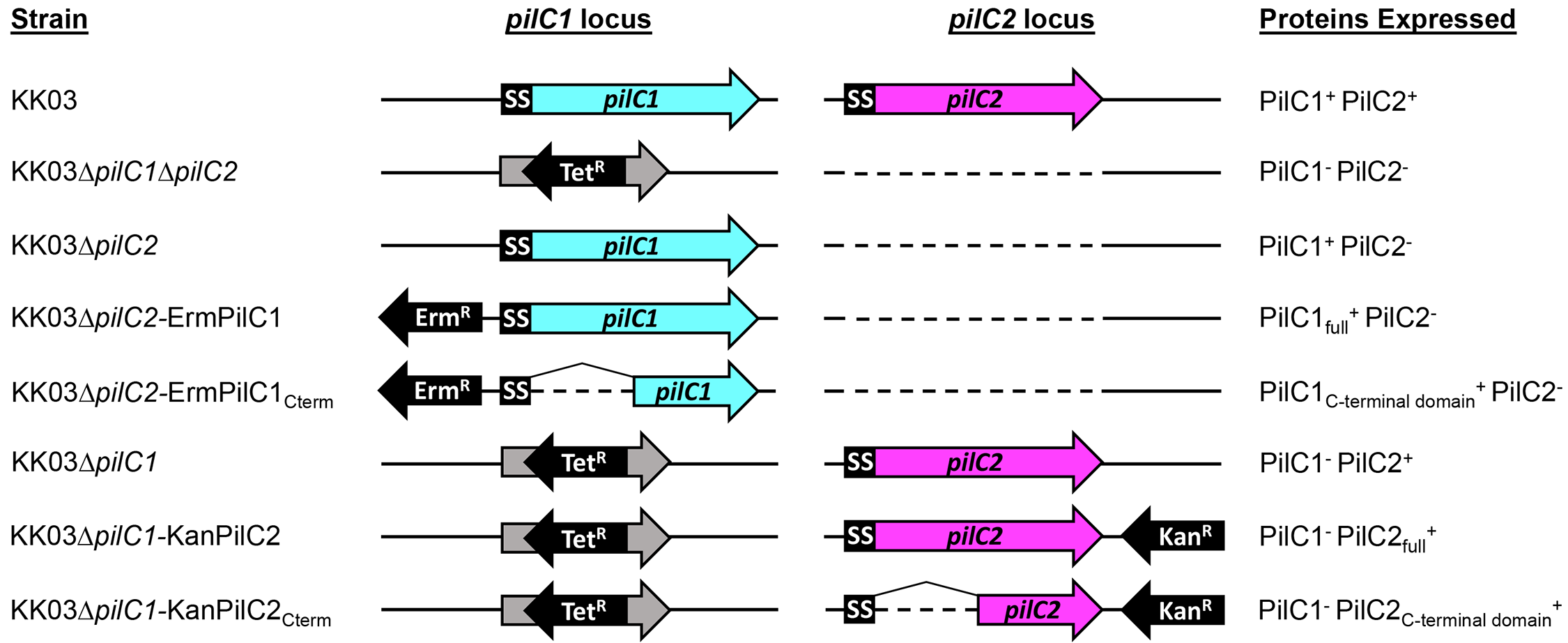

Supplement: S3 Fig — Strain KK03 (WT) produces PilC1 and PilC2, which are encoded by the pilC1 and pilC2 genes. Strain KK03ΔpilC1ΔpilC2 contains a tetracycline resistance cassette in place of pilC1 and an unmarked deletion of pilC2 and does not produce PilC1 or PilC2. Strain KK03ΔpilC2 contains an unmarked deletion of pilC2 and produces full-length PilC1. Strains KK03ΔpilC2-ErmPilC1 and KK03ΔpilC2-ErmPilC1Cterm contain an unmarked deletion of pilC2 and an erythromycin resistance cassette upstream of pilC1 and produce full-length PilC1 and the C-terminal domain of PilC1, respectively. Strain KK03ΔpilC1 contains a tetracycline resistance cassette in place of pilC1 and produces full-length PilC2. Strains KK03ΔpilC1-KanPilC2 and KK03ΔpilC1-KanPilC2Cterm contain a tetracycline resistance cassette in place of pilC1 and a kanamycin resistance cassette downstream of pilC2 and produce full-length PilC2 and the C-terminal domain of PilC2, respectively. SS represents the predicted signal sequence. Cyan color denotes strains producing either full-length PilC1 or the C-terminal region of PilC1; magenta color denotes strains producing either full-length PilC2 or the C-terminal region of PilC2. The dashed lines indicate a deletion. TetR, KanR, and ErmR indicate tetracycline, kanamycin, and erythromycin resistance cassettes, respectively. (TIF) [file ppat.1010440.s003.tif]
